# Enabling Accurate and Large-Scale Explicitly Correlated CCSD(T) Computations via a Reduced-Cost and Parallel Implementation

**DOI:** 10.1021/acs.jctc.4c01777

**Published:** 2025-02-26

**Authors:** Bence Ladóczki, László Gyevi-Nagy, Péter R. Nagy, Mihály Kállay

**Affiliations:** † Department of Physical Chemistry and Materials Science, Faculty of Chemical Technology and Biotechnology, Budapest University of Technology and Economics, Műegyetem rkp. 3., H-1111 Budapest, Hungary; ‡ HUN-REN-BME Quantum Chemistry Research Group, Műegyetem rkp. 3., H-1111 Budapest, Hungary; § MTA-BME Lendület Quantum Chemistry Research Group, Műegyetem rkp. 3., H-1111 Budapest, Hungary

## Abstract

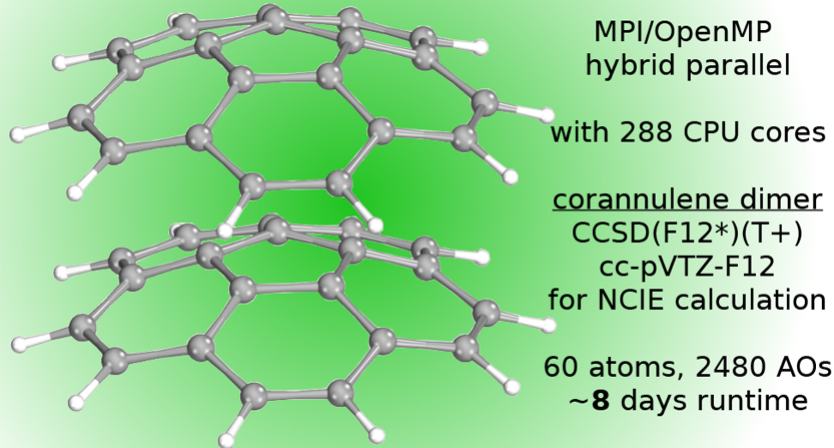

Parallel algorithms to accelerate explicitly correlated second-order Mo̷ller–Plesset (MP2) and coupled-cluster singles and doubles with perturbative triples [CCSD(T)] calculations and benchmarks on extended molecular systems are reported. A hybrid Open Multi-Processing (OpenMP)/Message Passing Interface (MPI) parallel approach is used to distribute the computational load among processor cores and compute nodes. The intermediates at both the MP2 and the CCSD(T) levels are expressed in a density fitting formalism, using only three-index quantities to decrease the amount of data to be stored and communicated. To further reduce compute time, the frozen natural orbital, the natural auxiliary function, and the natural auxiliary basis schemes are implemented in a hybrid parallel manner. The combination of these three approximations and our recent size-consistent explicitly correlated triples correction with the new hybrid parallelization offers a unique accuracy-over-cost performance among explicitly correlated CC methods. Our comprehensive benchmarks demonstrate excellent parallel scaling of the cost-determining operations up to hundreds of processor cores. As demonstrated on the noncovalent interaction energy of the corannulene dimer, highly accurate explicitly correlated CCSD(T) calculations can be carried out for systems of 60 atoms and 2500 orbitals, which were beyond computational limits without local correlation approximations. This enables various applications, such as benchmarking of or, for certain size ranges, replacing local CCSD(T) or density functional methods as well as the further advancement of robust thermochemistry protocols designed for larger molecules of ca. 20–50 atoms.

## 1 Introduction

Wave function based quantum chemical methods can be systematically converged toward results often matching the accuracy of experiments, at least when molecule size permits. In many cases, it is still challenging to produce sufficiently converged results in terms of both the one-particle basis set and the level of electron correlation treatment. Regarding the latter, the Mo̷ller–Plesset perturbation series, in particular, its popular second-order MP2 variant,^1^ and even more so the coupled-cluster (CC) wave function hierarchy are the method of choice.^2^ Especially, the CC model with single and double excitations (CCSD)^3^ and CCSD with perturbative triples corrections [CCSD(T)]^4^ offer reliable accuracy. However, the number of floating point operations (FLOPs) scales as *n*_o_^2^*n*_v_^4^ for CCSD and *n*_o_^3^*n*_v_^4^ for (T), with *n*_o_ and *n*_v_ denoting the number of correlated occupied and virtual orbitals, respectively. Consequently, even the most powerful high-performance computing (HPC) clusters cannot significantly extend the limits of conventional CCSD(T), which is currently around 25–30 atoms (1500 orbitals) with well-converged basis sets.^5^

Regarding the slow basis set convergence of such finite-basis expansions, one of the most established remedies is the explicitly correlated approaches,^6−8^ while promising alternatives such as the transcorrelated CC methods by Alavi, Kats, Ten-no and others^9,10^ as well as the density-based basis-set correction (DBBSC) proposed by Toulouse, Giner, and their co-workers^11^ are also emerging. For explicitly correlated methods, the conventional Slater-determinant expansions are augmented with special configurations explicitly containing the interelectronic distances. For that purpose, most modern explicitly correlated approaches use Slater-type geminal factors (F12),^12^ accurately describing the behavior of the wave function at both short and large interelectronic distances. Utilizing these ideas, several explicitly correlated MP2 (MP2-F12) variants have been proposed,^13−18^ and their extensions to the CCSD level have also matured.^19−27^ These days, the most widely used approaches include the CCSD-F12a and CCSD-F12b methods of Werner and co-workers,^23,24^ the CCSD(2)_F1̅2̅_ scheme of Valeev et al.,^25,26^ and the CCSD(F12*) approach of Hättig, Tew, and Köhn.^28^ The practical extension of explicit correlation to triple and higher excitations is still an open question. Although rigorous approaches exist,^29−32^ heuristic schemes based on the scaling of the (T) correction offer more efficient alternatives.^24,33^ Among these methods, our recent (T+) correction is probably the most theoretically justified as it has tackled the size-inconsistency issue of previous scaling schemes.^33^

Although these methods successfully decrease the basis set incompleteness error of CCSD(T), its expensive seventh-power scaling remains. Thus, considerable effort has also been invested in breaking down their computational costs. Relying on local correlation approximations, both closed- and open-shell systems with 100–200 atoms can now be treated with F12 methods,^34−38^ while our local natural orbital (LNO)^39−41^ implementation of DBBSC-CCSD(T) can scale up to 1000-atom proteins.^42^ However, there is a caveat to using local correlation methods: they can introduce computational overhead for smaller systems with only a few dozen atoms, and sometimes the local approximations may not be sufficient for high-precision computations. Additionally, one may want to test the reliability of local or other approximations against robust CCSD(F12*)(T+) references. To cover these scenarios, we developed reduced-cost CCSD(F12*)(T+) methods^43^ by combining the frozen natural orbital (FNO)^44−47^ approximation to compress the virtual molecular orbital (MO) space and the natural auxiliary function (NAF)^48^ scheme utilized for the compression of the auxiliary basis set required for the density fitting (DF) approximation. In addition, we also proposed a third approach, the natural auxiliary basis (NAB) scheme to decrease the size of the complementary auxiliary basis (CABS)^49,50^ needed for the resolution of the identity approximations. Here, we further advance these reduced-cost CCSD(F12*)(T+) methods via efficient parallelization.

Considering that growth in computational power is originating almost exclusively through parallelism, there is a constant need to improve quantum chemistry algorithms and tailor them to massively parallel computers containing ever more central processing units (CPUs) and often also to graphical processing units (GPUs).^51^ Extensive recent work has focused on the efficient parallelization of conventional, i.e., not explicitly correlated, CCSD(T) implementations.^5,52−63^ Compared to that, much less attention has been paid to the parallelization of explicitly correlated methods. The parallel implementation aspects of explicitly correlated MP2 calculations were first considered by Valeev and Janssen for an early variant of explicitly correlated MP2.^64^ A massively parallel MP2-F12 code was developed by Ten-no and co-workers, and its good parallel performance was demonstrated using more than 65,000 CPU cores.^65^ Concerning explicitly correlated CC theory, a massively parallel implementation of the CCSD(2)_F1̅2̅_ approach was reported by Valeev and co-workers for closed-shell molecules, and its strong scaling was demonstrated on various hardware architectures.^66^ A significant progress has also been made by Werner and co-workers, who developed efficient parallelized local CCSD(T) approaches based on the CCSD-F12a/b ansätze.^35,67^

Pushing the limits of conventional CCSD(T) calculations, we reported an integral-direct CCSD(T) implementation with excellent parallel scaling while retaining an outstanding peak performance utilization of 50–70%.^5^ Building on that, we developed a reduced-cost variant of this CCSD(T) algorithm^68^ utilizing the FNO and the NAF approximations, pushing the limits (without local approximations) to 50–75 atoms and above 2000 atomic orbitals (AOs) with accessible resources of 100–200 CPU cores. Here, we extend this FNO-CCSD(T) code to explicitly correlated FNO-CCSD(T) by introducing efficient parallelization for the parts required for F12 computations. In particular, we present a parallel implementation of the CCSD(F12*)(T+) model, utilizing the theoretically most complete CCSD(F12*) variant in combination with our advanced (T+) and FNO-NAF-NAB approaches. As a spinoff, a parallelized MP2-F12 code is also developed. We employ integral direct, DF-based, and hybrid Open Multi-Processing (OpenMP)/Message Passing Interface (MPI) algorithms to minimize potentially slow data communication and for high parallel efficiency throughout the computation of the DF integral, MP2-F12 pair energy, and F12-dependent CC terms.

This paper is structured as follows. First, in Section 2.1, we summarize the key aspects of explicitly correlated theories, including DF and the necessary list of integrals, and discuss the parallel implementation of MP2-F12. Then, algorithmic and parallel computational details of the FNO, NAF, and NAB approaches are provided in Section 2.2. In Section 2.3, we describe an MPI-parallel implementation of the F12-dependent CC intermediates. In Section 2.4, the details of the OpenMP parallelization are presented. We then assess the parallel scaling performance of the new algorithms in detail. Finally, we illustrate the limits and utility of the new CCSD(F12*)(T+) code with the interaction energy calculation of the corannulene dimer containing 60 atoms.

## 2 Theory and Implementation

The working equations of the CCSD(F12*)(T+) method are documented in the literature,^28,33^ therefore, we omit these details. In this work, the focus is on the parallel calculation of the MP2-F12 contribution as well as the necessary integrals and F12-dependent intermediates for a CCSD(F12*)(T+) calculation, that is, on the most time-consuming terms amenable to parallelization. The parallelization of the solution of the CCSD(F12*) equations and the computation of the (T+) correction is not discussed here since the difference with respect to conventional CCSD and (T) calculations are small, and the parallelization of the latter was presented previously.^5^

In the ensuing sections, the relevant expressions are given in terms of spin orbitals. Our index convention is presented in Table 1.

**Table 1:** Notation for the Various Orbital Spaces

symbol	definition
*i*, *j*	correlated occupied orbitals
*o*	frozen core and correlated occupied orbitals
*a*, *b*	Hartree–Fock (HF) virtual orbitals
*p*, *q*	general HF orbitals (occupied, virtual)
*a′*, *b′*	CABS virtual orbitals
*p′*, *q′*	general orbitals (general HF, CABS virtual)
*P*, *Q*	DF auxiliary basis functions

Indices {*i*} will represent a block of occupied orbitals assigned to a particular MPI process. When {*i*} is used to index an intermediate, it shall imply that the corresponding elements of the intermediate are processed by a certain MPI process. The similar holds for {*ij*}, standing for a block of index pairs assigned to a particular process.

### 2.1 MPI-Parallel Calculation of the MP2-F12 Contribution

A CCSD(F12*)(T+) calculation commences with the calculation of the MP2-F12 energy. Thus, in what follows, we first revisit the most important parts of the MP2-F12 formalism and discuss its parallel implementation. We rely on ansatz 2B, the F + K commutator approximation, and the fixed amplitude approximation.^14,18,69^ The expression for the F12 correction to the MP2 energy, *E*^F12^, reads as
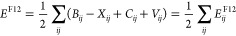
1The concrete equations for the four intermediates in the middle are presented elsewhere.^33^

When rewriting an existing sequential implementation of MP2-F12, it is enough to keep in mind that these terms are combinations of the matrix elements of the *g*_12_, *f*_12_, (∇̂_1_*f*_12_)^2^,  and *f*_12_/*r*_12_ operators. Here, *g*_12_ = 1/*r*_12_ and  with *r*_12_ denoting the interelectronic distance and γ an exponent. Observe that in eq 1, the summation runs over the *i* and *j* occupied indices, and therefore this formula lends itself to a convenient parallel implementation by distributing the required tasks along the pairs of occupied orbitals. The result for each MPI process is simply a scalar, and the data communication in this step is negligible. On the other hand, note that when the occupied space is not large enough this can lead to decreased parallel performance when the available number of compute nodes is large. However, in this case, the overall runtime is also lower, thus, a large number of MPI processes is not required.

The module that calculates the MP2-F12 energy stores only three-center matrix elements of the above operators, and it calculates the four-center integrals on the fly using DF.^18,70,71^ The four-center electron repulsion integrals, in the (11|22) convention, are approximated as
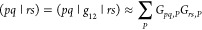
2and the integrals of the *f*_12_ correlation factors are evaluated using robust fitting formulas as follows:
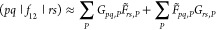
3In the above expressions, the following definitions are used for the fitting coefficients:
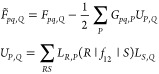
4
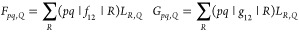
5where *L*_*R*,*P*_ are the elements of the lower triangular Cholesky-matrix obtained by decomposing the inverse of the two-center Coulomb integral matrix (*P*|*Q*). The *f*_12_ kernel can also be replaced by the rest of the above-mentioned operators to generate lists of (∇̂_1_*f*_12_)^2^, , and *f*_12_/*r*_12_ integrals. These will be denoted by **D**, **S**, and **R**, respectively. Note that the calculation of **F** and the latter lists necessitates **G**, therefore, when **G** is calculated for *g*_12_, it has to be stored so that it can be reused for the calculation of the rest of the integral lists.

**Figure 1:**
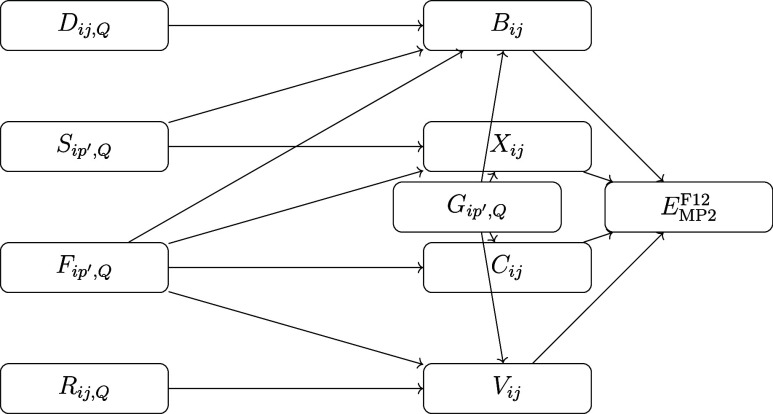
Dependency graph of an MP2-F12 calculation. Note that while different intermediates depend on different lists of integrals, every intermediate requires *G*_*ip′*,*Q*_ and *G*_*jp′*,*Q*_ due to robust DF.

The data dependency of the MP2-F12 energy in terms of the three-center integrals is illustrated in Figure 1, which was constructed using the formulas for *B*_*ij*_, *X*_*ij*_, *C*_*ij*_, and *V*_*ij*_ as given in ref (33). For the sake of clarity, two-center integrals are omitted from the graph. For an MP2-F12 calculation, the *G*_*ip′*,*Q*_, *F*_*ip′*,*Q*_, *D*_*ij*,*Q*_, *S*_*ip′*,*Q*_, and *R*_*ij*,*Q*_ blocks of the above intermediates are needed. When one wishes to perform a CC calculation as well, the *G*_*pq′*,*Q*_, *F*_*ip′*,*Q*_, and *R*_*ip*,*Q*_ lists are necessary for the construction of the F12-dependent intermediates on the CC level.

When calculating contributions to *E*^F12^ from *B*_*ij*_, *X*_*ij*_, *C*_*ij*_, and *V*_*ij*_ employing eq 1, the four-center integrals are evaluated according to eqs 2 and 3 using the above three-index intermediates. The explicitly correlated MP2 energy can then be calculated from the appropriate contractions of the integral lists following the prescription dictated by the working equations of *E*^F12^.

In the current implementation, the last index (column-major order) of the arrays storing the three-center integrals *G*_*ip′*,*Q*_, *F*_*ip′*,*Q*_, *D*_*ij*,*Q*_, *S*_*ip′*,*Q*_, and *R*_*ij*,*Q*_ represents occupied orbitals *i*. For *G*_*pq′*,*P*_, when CC intermediates are generated after the MP2-F12 calculation, the last index is a general HF MO index *p*. For *F*_*ip′*,*Q*_, *S*_*ip′*,*Q*_, and *R*_*ij*,*Q*_ this last index is split up, the calculation of each term is distributed among the MPI processes, and the terms are assembled for every MPI process by calls to MPI library functions. Note that this broadcast operation can be avoided for *D*_*ij*,*Q*_, because the energy contribution can be calculated for the occupied indices independently. In this case, only the correlation energy contribution is collected rather than the entire integral list. This intermediate contributes to the correlation energy via the term , where *Ŝ*_*ij*_ = 3/8 + 1/8*P̂*_*ij*_, and *P̂*_*ij*_ permutes the spatial components of spin orbitals *i* and *j* in determinant |*ij*⟩. This term can be evaluated from three-index fitting coefficients by computing the matrix *D*_*ij*_ = ∑_*Q*_*G*_*ij*,*Q*_*D*_*ij*,*Q*_ while paying attention to the permutation of the indices. The contribution of *D*_*ij*_ to *B*_*ij*_ in a restricted range {*j*} can be written formally as follows:
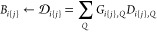
6As such, the contribution to *B*_*ij*_ can be calculated without broadcasting the integral list *D*_*ij*,*Q*_. In practice, we initialize an empty array for the contribution at every MPI process, and once the contribution is calculated by the processes, a parallel summation (MPI_Allreduce) is performed. Note that the size of this array is much smaller than the size of *D*_*ij*,*Q*_, and this incurs negligible communication overhead.

Based on these observations, we designed Algorithm 1 for the evaluation of the MP2-F12 pair energies. The loops shown in the scheme are all MPI-parallel ones. The calculation starts with the parallel evaluation and assembly of the intermediate **G**. Once this is done, the full **G** is stored in memory for each MPI process during the rest of the calculation. Then, **D** and the corresponding correlation energy contribution are evaluated in parallel. **D** is neither broadcast nor stored. In the next step, intermediate **R** is computed in parallel and broadcast to each process. Its contribution to the pair energies is evaluated, and then, it gets discarded if no CC calculation is performed. Thereafter, **S** is calculated and processed in the same way as **R**. The only difference is that **S** is never stored beyond this point as it is not needed for the CC intermediates. Finally, intermediate **F** is computed in parallel, broadcast to all MPI processes, and its energy contributions are calculated. Should one calculate a CC wave function, **F** is retained.
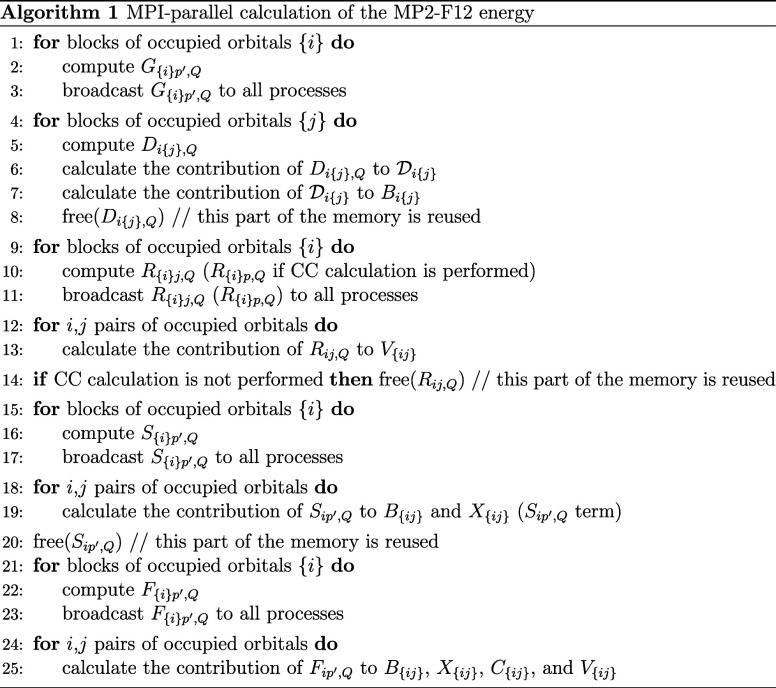


**Figure 2:**
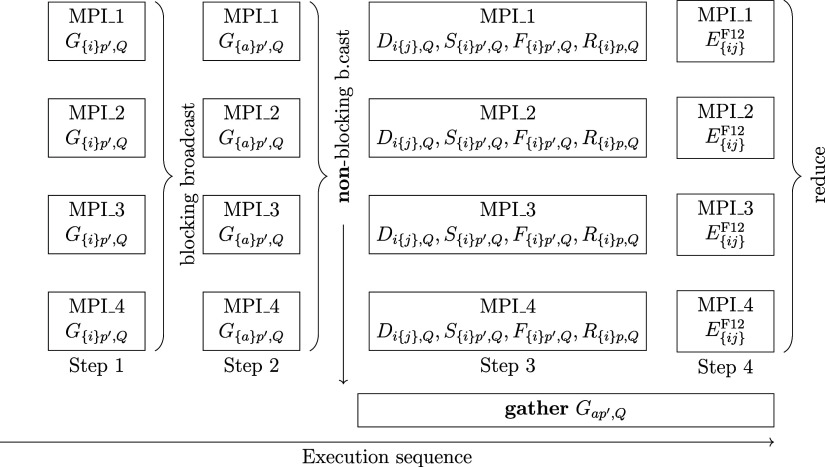
Schematic illustration of the time horizon of an MP2-F12 calculation with an efficient parallel communication of *G*_*pq′*,*Q*_ for 4 MPI workers. The extension to more workers is trivial. Notice that the collection of the *G*_*aq′*,*Q*_ block takes place when the other integrals and the MP2-F12 energy are calculated as there is no data dependency between these steps.

The computation of the MP2-F12 pair energies requires only *G*_*ip′*,*Q*_-type integrals. When a CC calculation is also carried out after the MP2-F12 step, *G*_*ap′*,*Q*_-type fitting coefficients are also necessary. In this case, one of the most time-consuming steps is the calculation and, in particular, the collection of the entire *G*_*qp′*,*Q*_ integral list. To achieve better parallel efficiency, the communication of its virtual block can be performed asynchronously as illustrated in Figure 2. The *G*_*ip′*,*Q*_ block of the integral list is computed at the beginning and collected using blocking broadcast calls. Then, the remaining virtual block is evaluated, but it is collected using a nonblocking broadcast during the calculation of the MP2-F12 energy. The successful termination of the gather operation is only checked when the execution reaches the calculation of the CC intermediates. The *G*_*ap′*,*Q*_ block of *G*_*qp′*,*Q*_ is usually much larger than *G*_*ip′*,*Q*_, thus the nonblocking collection of data can save significant time, and this will be demonstrated in Section 3 down below.

### 2.2 FNO-NAF-NAB Approach with MPI

If a reduced-cost CCSD(F12*)(T+) calculation is performed utilizing the FNO, NAF, and NAB approximations, the corresponding orbital spaces are constructed before computing the F12-dependent CC intermediates.^43^ Though these operations are relatively cheap, they are also parallelized as without parallelization, they may become the bottleneck with very compact FNO and NAF spaces and a large number of compute cores.

The FNO approach enables one to represent the wave function in a more compact form. To this end, the MP2 one-particle density matrix is required, which can simply be calculated in parallel as a byproduct of the computation of intermediate *C*_*ij*_. It is then diagonalized to obtain its eigenvalues and the corresponding eigenvectors, that is, the natural orbitals (NOs). The diagonalization is always performed on the main MPI process to ensure that all NOs have the same sign (phase). The eigenvalues that are smaller than a threshold (*t*_FNO_) are discarded along with the corresponding NOs. Subsequently, the corresponding indices of intermediates **G**, **F**, and **R** are transformed to the truncated NO basis.

The time-consuming transformation of *G*_*qp′*,*Q*_ to the FNO basis is MPI-parallelized. Note that this list of integrals has two indices that cover the virtual space, therefore, the MPI-parallel transformation is performed in two steps. First, index *q* of *G*_*qp′*,*Q*_ is scattered among the MPI processes, and the HF virtual index range of *p′* is transformed. The fragments of *G*_*qp′*,*Q*_ are collected and the full matrix is broadcast to all MPI processes. As the size of the transformed *G*_*qp′*,*Q*_ is still comparable to that of the original one, this step incurs a significant communication overhead. Second, *p′* is split up, and the virtual index range of *q* is transformed, followed by a parallel summation.

The virtual indices of *F*_*ip′*,*Q*_ and *R*_*ip*,*Q*_ are also transformed to the FNO space in a parallel manner. Owing to the fact that only the *p′* and *p* indices, respectively, run over the virtual orbitals, and these are the first indices (column-major order), the situation is much less complicated than for *G*_*qp′*,*Q*_. For *F*_*ip′*,*Q*_ and *R*_*ip*,*Q*_, the slower indices, *i* and *Q* are used to create hyperindices, and these are distributed among the MPI processes. For each process, the FNO transformation is performed on the virtual indices, and the transformed integral lists are broadcast, which takes much less time than the broadcast of *G*_*qp′*,*Q*_.

Due to the truncation of the HF virtual MO space, the coupling of the explicitly correlated excitations and those conventional excitations for which the excitation would land on a dropped virtual NO is missing. We approximate this missing contribution at the MP2-F12 level.^43^ In practice, the entire coupling contribution, that is, intermediate *C*_*ij*_ is evaluated in the original MO basis together with the MP2-F12 energy as described above. To compute the correction, *C*_*ij*_ is also calculated in the truncated MO basis analogously to the MP2-F12 computation in the complete virtual MO basis.

In the next step, the functions of the CABS are combined to form the NAB space, following a process similar to the construction of the FNOs.^43^ To that end, the procedure starts with the parallelized construction of the matrix *W̅*_*a′b′*_ = ∑_*p*,*P*_*G*_*pa′*,*P*_*G*_*pb′*,*P*_. Here, hyperindices formed from the summation indices are distributed among the MPI processes, and the resulting contributions to *W̅*_*a′b′*_ are reduced. Note that this does not incur a large communication overhead since the size of the matrices to be communicated is equal to the square of the CABS virtual space. Subsequently, the matrix is diagonalized, and the resulting NAB space is truncated. At the end, the CABS virtual indices of matrices *G*_*qp′*,*Q*_ and *F*_*ip′*,*Q*_ are transformed to the NAB eigenspace using a similar approach as for the FNO method.

Finally, the DF auxiliary basis is compressed by creating NAFs. This is achieved by first constructing the matrix *W*_*PQ*_ = ∑_*q*,*p′*_*G*_*qp′*,*P*_*G*_*qp′*,*Q*_. The matrix is assembled using MPI processes running over the summation indices and the resulting contributions to *W*_*PQ*_ are reduced. The size of the disseminated matrices is small, equal to the square of the size of the auxiliary basis, and they can be collected in negligible time. Once constructed, the matrix is diagonalized, and the NAF eigenspace is truncated. Next, the DF auxiliary index *Q* of intermediates *G*_*qp′*,*Q*_, *F*_*ip′*,*Q*_, and *R*_*ip*,*Q*_ is transformed to the NAF basis. In the current implementation, the DF auxiliary index is always in the middle, that is, the second slowest in column-major order. The last index, *q* for *G*_*qp′*,*Q*_ and *i* for the other two intermediates, can be used to distribute the computation load among the MPI processes. By using such an organization of indices, the results can be gathered in a trivial manner.

### 2.3 MPI Parallelization of the F12-Dependent CC Intermediates

For the MPI-parallel implementation of CCSD(F12*)(T+), we leverage our highly optimized conventional DF-CCSD(T) code^5,68^ and extend it with MPI-parallel F12-dependent intermediate terms relying similarly on DF. The corresponding CC intermediates within the fixed amplitude approach, , , , , , and , are available in the literature.^28^ We will only review the expressions essential to our parallel implementation.

**Figure 3:**
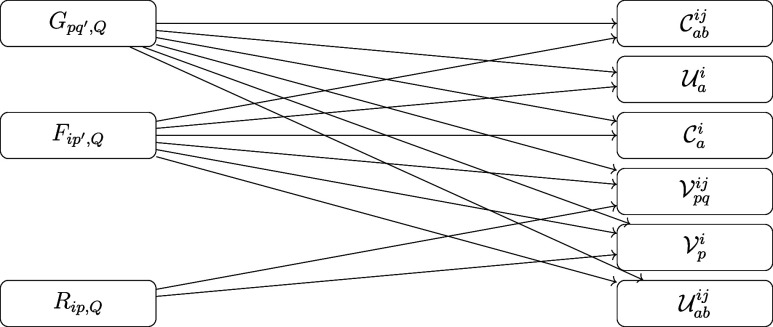
Data dependency graph of the explicitly correlated CC intermediates. *G*_*pq′*,*Q*_ with one DF auxiliary index (*Q*), one general index (*q′*) and one general HF MO index (*p*) dominates the memory requirement of this step.

The data dependency of these CC intermediates is illustrated in Figure 3.

The most time-consuming intermediate term in a CCSD(F12*) calculation is :
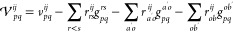
7which is constructed from the following tensors:
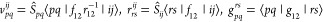
8The computational cost of this term is dominated by the second, third, and fourth terms on the right side of eq 7.

There are three groups of indices in eq 7, *a′o* or *rs*, *pq*, and *ij*, that can be used to create hyperindices and to split them up among MPI processes. Each of these options leads to a different algorithm and involves varying amounts of communication overhead. Notice that *a′* is a CABS virtual index, *o* is an occupied index, *ij* are correlated occupied indices, while *pq* and *rs* are general MO indices in the conventional HF basis, which implies that **g** is by far the largest quantity throughout a CCSD(F12*) calculation. It scales roughly with the fourth power of the AO basis set size, hereafter denoted by *n*_b_. For this reason, **g** in its entirety cannot be stored in memory even for small systems, and this necessitates a loop over its blocks. It seems reasonable to distribute either the summation or the *pq* index pairs to MPI processes. We will adopt the latter approach; however, let us first briefly elucidate the drawback of parallelizing the summation. For example, for the second term, MPI parallelization over the *rs* summation index pair could be implemented as
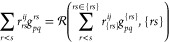
9where {*rs*} stands for the pairs of indices allocated to a certain MPI process, and the restriction on the summation indicates that it is performed only for those index pairs for which *rs* is allocated to the process. In the above equation,  denotes a formal MPI reduction operator (i.e., MPI_Allreduce), which reduces its first argument for the index range specified by its second argument. In this way, each process would generate intermediate tensors of size ∼ *n*_o_^2^*n*_b_^2^. To address this, one would need to immediately gather them through extensive communication operations or store them and reduce them at the end of the loop over the blocks of **g**.

In comparison, distributing the *pq* index pairs among MPI processes is far more beneficial. Due to the memory bottleneck of storing **g**, it can only be calculated from *G*_*pq*,*Q*_ in blocks. Splitting this up over the *pq* index pairs solves both the storage bottleneck and MPI-parallel load distribution issues. The size of the blocks is determined by the memory space that is available for the calculation. The objective here is to exhaust the remaining available memory and to process arrays that are as large as possible. In the parallel implementation, the *pq* indices can be distributed to MPI processes so that every process calculates a block of . For *N* MPI processes, this approach reduces the memory requirement for the storage of **g** by a factor of *N* (assuming one MPI process per node). Another gain is that such a parallel implementation entails a much smaller communication overhead because obviously, a block is always smaller than the entire . Finally, one could consider distributing the occupied index pairs *ij*, but the occupied space is usually much smaller and this would not help with the storage bottleneck associated to **g**. Therefore, we scatter the general HF MO index pair *pq* among the MPI processes, and thus intermediate  is evaluated as
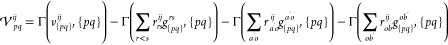
10where Γ denotes a formal MPI communication operator (i.e., MPI_Allgatherv).

The contribution ∑_*r*<*s*_*r*_*rs*_^*ij*^*g*_*pq*_^*rs*^ is very similar to the particle–particle ladder (PPL) term of conventional CCSD equations. Accordingly, the algorithms elaborated for the PPL term can be adopted here, which results in significant savings in the closed-shell case. Then, this term reduces to ∑_*rs*_*r*_*rs*_^*ij*^⟨*pq*|*rs*⟩, where the indices now stand for spatial orbitals, and ⟨*pq*|*rs*⟩ is a four-center integral in the ⟨12|12⟩ convention. The term can be tackled by recasting it as a sum of symmetrized and antisymmetrized contributions as^72−74^
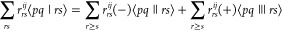
11where *r*_*rs*_^*ij*^(−) and *r*_*rs*_^*ij*^(+) are
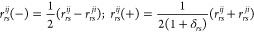
12The antisymmetrized and symmetrized two-electron integrals are defined, respectively, as
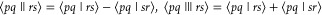
13⟨*pq*||*rs*⟩ and ⟨*pq*|||*rs*⟩ are available to every MPI process as *G*_*pq*,*Q*_ is replicated to each one. The PPL-like contractions are performed by parallel MPI processes, where the *pq* indices run over a range confined to the actual process, i.e., only ∑_*r*≥*s*_*r*_*rs*_^*ij*^(−)⟨{*pq*}||*rs*⟩ and ∑_*r*≥*s*_*r*_*rs*_^*ij*^(+)⟨{*pq*}|||*rs*⟩ are calculated, and these contributions are finally reduced as described above for the general case.

The parallel assembly of the rest of the terms is relatively straightforward as the summations can be performed independently. For example, the term  is calculated as
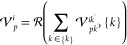
14The construction of  can be carried out analogously, and we omit these less interesting details. Next, we evaluate  and  together as their sum is needed for the CCSD iteration. They have a similar structure, and both depend on *f*_12_ integrals:^28^
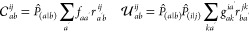
15where *f*_*aa′*_ stands for an element of the Fock matrix, and *P̂*_(*a*|*b*)_ is an antisymmetrizer operator; e.g., *P̂*_(*a*|*b*)_*f*_*ab*_ = *f*_*ab*_ – *f*_*ba*_. In our MPI parallel implementation every MPI process calculates a block of  split up over one of its occupied index, and the same index is used for the summation in the calculation of  Then, the resulting arrays are summed via an MPI communicator (MPI_Allreduce) and saved into a file to be used within the CCSD iterations.

### 2.4 OpenMP Parallelization

We combine MPI with shared memory OpenMP thread parallelism for the time-consuming terms to reduce data storage and communication compared to an MPI-only implementation. The general idea is that the outer loops are parallelized with MPI, while the inner loops are parallelized with OpenMP. This structure is beneficial for current HPC clusters, where several interconnected nodes are furnished with multiple CPUs usually featuring many computing cores and ever-shrinking memory-per-core resources. At the OpenMP level, whenever possible, vectorized and threaded level 3 Basic Linear Algebra Subprograms (BLAS3) calls are prioritized, e.g., by performing matrix–matrix multiplications via dgemm routines. When this is not possible, we implement the outermost loops that are not MPI parallelized using OpenMP directives.

In more detail, first, the two- and three-center integrals are calculated using a general integral evaluator module, which also transforms one of the AO indices to the HF MO basis.^33^ Here, dynamically scheduled OpenMP is used for the loop over the atoms on which the fitting functions reside. As explained above, four-index quantities are never stored, they are directly assembled via DF formulas using thread-parallel matrix–matrix multiplications (via dgemm). The pair energies *E*_*ij*_^F12^ and F12-dependent intermediate terms to CCSD are calculated in a similar way, whenever possible, using thread-parallel matrix–matrix multiplications for large blocks determined by the MPI processes (and memory bottlenecks). The remaining parts contain summations with arrays available in the shared memory space, thus their OpenMP parallelization is relatively simple and not discussed in detail. Considering the FNO, NAB, and NAF bases, their construction and the corresponding integral transformations can be implemented using thread-parallel BLAS3 and Linear Algebra Package (LAPACK) routines.

All in all, especially for the most time-consuming PPL-like terms, this hybrid approach efficiently combines the benefits of integral-direct four-center integral assembly, communication-economic shared memory parallelization via OpenMP for the data intensive parts, and well-scalable MPI strategies for the operation intensive parts.

## 3 Results

### 3.1 Computational Details

The parallelized CCSD(F12*)(T+) algorithm presented has been implemented in the Mrcc quantum chemistry suite,^75,76^ which was also used in the calculations discussed herein. The employed molecular structures can be found in the Supporting Information (SI).

The correlation consistent *X*-tuple-ζ (*X* = D, T, Q) AO basis sets designed for explicitly correlated calculations (cc-pV*X*Z-F12)^77^ were employed together with the corresponding cc-pV*X*Z-F12-OPTRI CABS bases.^78,79^ The DF approximation was utilized both at the HF and the correlated levels utilizing the aug-cc-pV(*X*+1)Z-RI-JK^80^ and the aug-cc-pwCV(*X*+1)Z-RI^81^ auxiliary basis sets, respectively. The frozen core approximation was invoked in all the correlation calculations.

The computations were conducted on an HPC architecture powered by dual AMD EPYC 7763 64-core processors (2 physical CPUs per node) and 256GB of memory per node composed of DDR4 memory modules at 3200 MHz with a capacity of 16GB each. The HPC system that we utilized is equipped with the HPE Slingshot 200GbE interconnect (one card per node), which provides 25.6 Tb/s of bidirectional bandwidth. The computations on the HPC nodes were performed with the following settings: OMP_PLACES = cores, OMP_PROC_BINDS = close, I_MPI_PIN = on, and I_MPI_PIN_DOMAIN = *p*_d_:compact, where *p*_d_ denotes 2× the number of physical cores to avoid hyperthreading for the case when only one MPI process was used per node.

### 3.2 Nonblocking Broadcast

**Figure 4:**
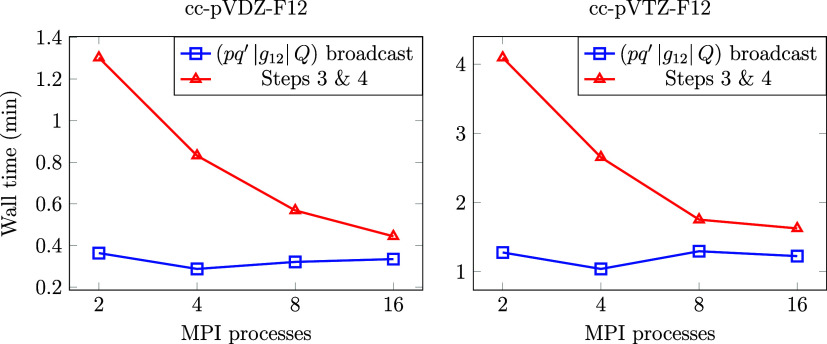
Time required to gather the virtual block of (*pq′*|*g*_12_|*Q*) and the time required for Steps 3 and 4 (see Figure 2) in minutes. Results using the anthracene molecule (66 active electrons) with the cc-pV*X*Z-F12 (*X* = D, T) basis sets are presented.

First, we measure whether the communication of the virtual block of (*pq′*|*g*_12_|*Q*) can be successfully hidden behind the MP2-F12 energy evaluation steps (Steps 3 and 4, as explained in Figure 2) using nonblocking broadcast. This is not obvious as (*pq′*|*g*_12_|*Q*) can be of very large size, and its communication time is roughly constant or even increasing with the number of MPI processes, while the time for Steps 3 and 4 of MP2-F12 decreases efficiently with the number of MPI processes. To quantify this, we run calculations with MPI processes executed on physically separated nodes and inspect the results of our wall clock time measurements using the cc-pVDZ-F12 and cc-pVTZ-F12 basis sets for the anthracene (C_14_H_10_) molecule^82^ in Figure 4. We find that the performance of the nonblocking broadcast implementation with the high-quality network employed is satisfactory already for this relatively small molecule. As the number of operations for MP2-F12 scales more steeply with the system size than the size of (*pq′*|*g*_12_|*Q*), in practice, one can expect the MP2-F12 computation to take long enough to cover the (*pq′*|*g*_12_|*Q*) broadcast, even with a large number of MPI processes.

### 3.3 MPI Parallel Efficiency of the F12 Terms

Next, we evaluate the parallel performance of our implementation focusing on the above-introduced lists of integrals, MP2 energies, and CC intermediates. According to our experience, the calculation of the three-index Coulomb integrals consumes significant CPU time. On the other hand, the rest of the integral lists, the coupling term, and the MP2 pair energies are less costly. Regarding the CC intermediates,  and  are both very expensive, and depending on the molecule and the basis set the calculation of  can also be lengthy. A comprehensive examination of MPI speedups was performed on a cyclic dihydrooxazine N-oxide (abbreviated as OO) molecule^83^ with the cc-pVDZ-F12 basis using MPI processes that are physically separate from each other. For this 40-atom system, with 108 active electrons and 760 active atomic orbitals, the size of (*pq′*|*g*_12_|*Q*) takes up roughly 52 GB of memory, and the integral lists with all the necessary terms require about 121 GB of memory.

**Figure 5:**
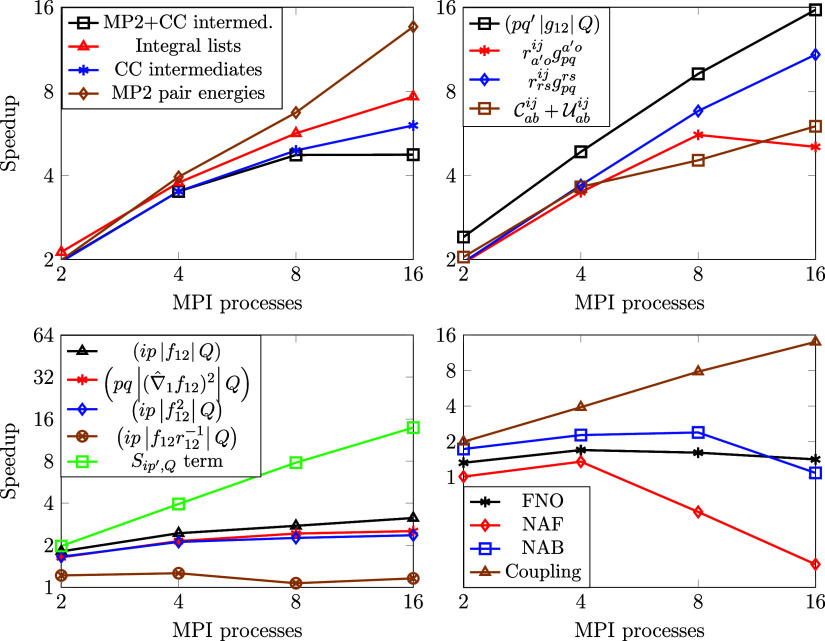
Speedup wrt. the number of MPI processes (8 OpenMP threads per process) for the OO molecule in the cc-pVDZ-F12 basis (108 active electrons, 760 active AOs) utilizing the FNO, NAF, and NAB techniques.

We analyze the speedup of different terms from 1 up to 16 MPI processes invoking Figure 5. The scaling properties of the major steps are displayed in the top-left subplot, including the total runtime (“MP2+CC intermed.”). A detailed breakdown of the speedups for the various operations is presented in the other subplots using the notation of Section 2.

The pair energies exhibit the best parallel efficiency as there is no expensive data transfer in this step. This can be attributed to the fact that the outermost loops that run over occupied orbitals are distributed among MPI processes, and the result of this step is of small size. The computationally most demanding terms among the CC intermediates also scale well because the computational load is sufficiently large, while the result to be communicated is relatively small. Note on the top-right panel that the largest integral list, (*pq′*|*g*_12_|*Q*), exhibits almost ideal scaling. This is due to the fact that the computational load is large, and it can be efficiently distributed among the MPI processes (and its communication is efficiently hidden behind the operation intensive steps, c.f., Section 3.2).

For completeness, we also inspect the remaining parts, which are, however, far from being rate-determining. The scaling of each individual integral list is presented in the bottom left subplot of Figure 5, while the FNO, NAF, and NAB transformations are shown in the bottom-right. Except for the *S*_*ip′*,*Q*_ and coupling terms, the MPI parallel scaling of these parts is far from ideal. This can be at least partly explained by the fact that these operations manipulate and communicate large matrices, e.g., involving the permutation of indices and/or other extensive memory operations, which are known to scale poorly. Since the parallel efficiency somewhat improves with increasing molecule size (c.f., penicillin^84^ in the SI), and these parts take just a few percent of the total runtime, at the moment, there is no motivation for their further optimization.

**Figure 6:**
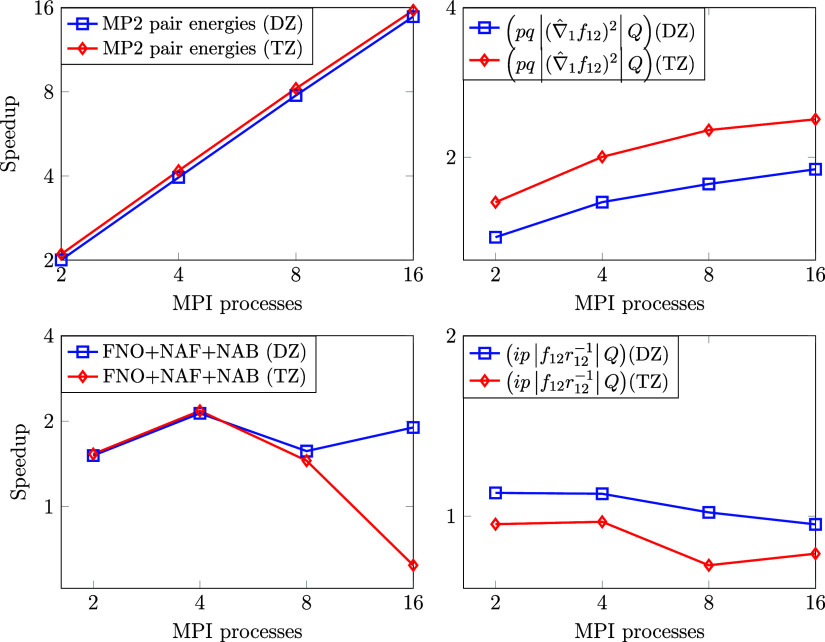
Speedup wrt. the number of MPI processes (8 OpenMP threads per process) for the anthracene molecule (66 active electrons) using the cc-pV*X*Z-F12 (*X* = D, T) basis sets (496 and 908 AOs, respectively) utilizing the FNO, NAF, and NAB techniques.

It is also instructive to enlarge the basis set and investigate the extent up to which one can accelerate the calculation of the MP2-F12 correlation energy and the CC intermediates using MPI. The speedups of the representative steps are presented in Figure 6 for the anthracene molecule using both the cc-pVDZ-F12 and the cc-pVTZ-F12 basis sets. A larger AO basis implies a larger DF basis, more virtual orbitals and hence higher operation count for the terms that depend on these dimensions. The top-left plot of Figure 6 shows close to ideal acceleration similarly for both basis sets. Regarding (*pq*|(∇̂_1_*f*_12_)^2^|*Q*) (top-right), the scaling is notably better with the larger basis set, as expected, due to the increased computational load. In contrast, the  term scales worse with the larger basis set (bottom-right plot). This can be understood by recalling that  is always collected because a CC calculation necessitates the entire intermediate. This means that considering our current implementation, the larger it grows, the worse its parallel efficiency becomes. For completeness, the bottom-left plot shows similarly poor scaling for the FNO, NAF, and NAB transformation with both basis sets. The rate-limiting step here is the parallel reduction (MPI_Allreduce) of the virtual block of (*pq′*|*g*_12_|*Q*) in its transformed form. The parallel gain diminishes as we increase the number of processes to about 8–16. As noted above, the FNO transformation takes only a few percent of the entire wall time, not even speaking of the evaluation of the (*ip*|*f*_12_|*Q*), (*pq*|(∇̂_1_*f*_12_)^2^|*Q*),  and  tensors, which are usually much smaller than (*pq′*|*g*_12_|*Q*) (as shown, e.g., in terms of relative timings below).

### 3.4 Overall MPI Scaling of CCSD(F12*)(T+)

The scaling with respect to the number of MPI processes of entire explicitly correlated CCSD(F12*)(T+) calculations using the FNO-NAF-NAB approximations is benchmarked on 5 molecules of 28–42 atoms, using 8 CPU cores per MPI process. The systems were chosen so that the memory requirement of the calculation does not exceed a single node’s memory capacity (256 GB) in our cluster. The largest tensor in our calculations is the 3-center Coulomb integral fitting coefficient tensor, whose size scales with the size of the HF orbital space plus the CABS space, the DF auxiliary function space, and the HF orbital space. The total memory requirement of CCSD(F12*)(T+) varies between 60–180 GB for these examples.

**Figure 7:**
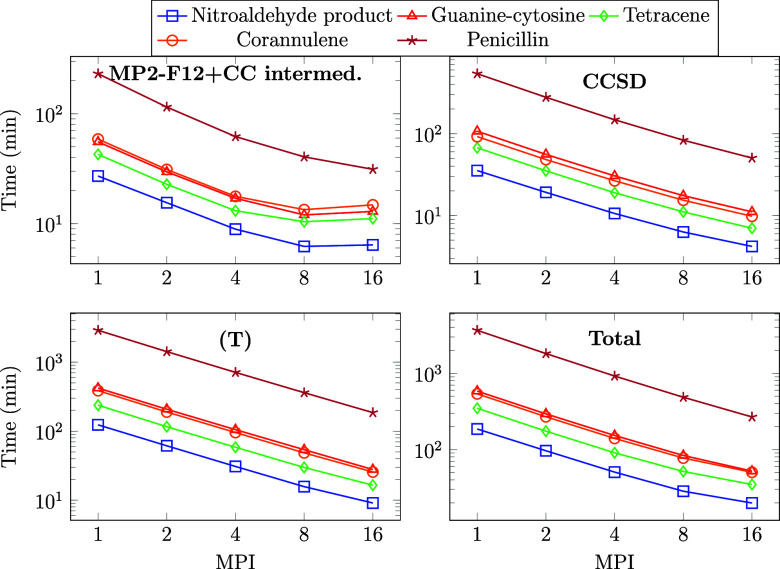
Wall times of explicitly correlated CCSD(T) calculations in minutes for 5 molecules (of 28–42 atoms) in the cc-pVDZ-F12 basis set with respect to the number of MPI processes (physically separated nodes). The abbreviation “MP2-F12+CC intermed.” stands for the calculation of all the necessary integrals; the MP2-F12 energy; the FNO, NAB, and NAF transformations; and the calculation of the F12-dependent CC intermediates.

The total computation times as well as the separate timings for the MP2-F12 plus CC intermediate, CCSD, and (T) calculations are collected in Figure 7 with the detailed timing data shown in Table S1 of the SI. We achieved the best total scaling of 13.7 for the penicillin molecule with 42 atoms (the largest one in this benchmark) with 16 MPI processes. Note that the sequential calculation took roughly 2.5 days, while it required only 4.5 h to obtain the explicitly correlated CCSD(T) energy when we utilized 16 MPI processes.

The CCSD and the (T) calculations exhibit better MPI scaling than the calculation of the F12 integrals and the CC intermediates. This is attributable to the fact that both the FNO-NAF-NAB transformations and the calculation of the integrals entail significant communication overhead. Nonetheless, we find good MPI scaling also for these F12-dependent parts up to 4–8 MPI tasks for the smaller systems, while the speedup values plateau somewhat later for larger molecules, e.g., beyond 16 MPI processes for penicillin. More specifically, the speedup of the F12-dependent parts (see Figure 7 and Table S1 of the SI) from 1 to 16 MPI processes is 4.2 (7.4) for the 28-atom nitroaldehyde product (42-atom penicillin). Compared to that, the better scaling and considerably longer runtime of the CCSD and (T) parts lead to roughly twice as good scaling of the wall time for the entire CCSD(F12*)(T+) computation, that is, 9.3 (13.7) with 16 MPI tasks.

### 3.5 Scaling of the Hybrid OpenMP and MPI Approach

**Figure 8:**
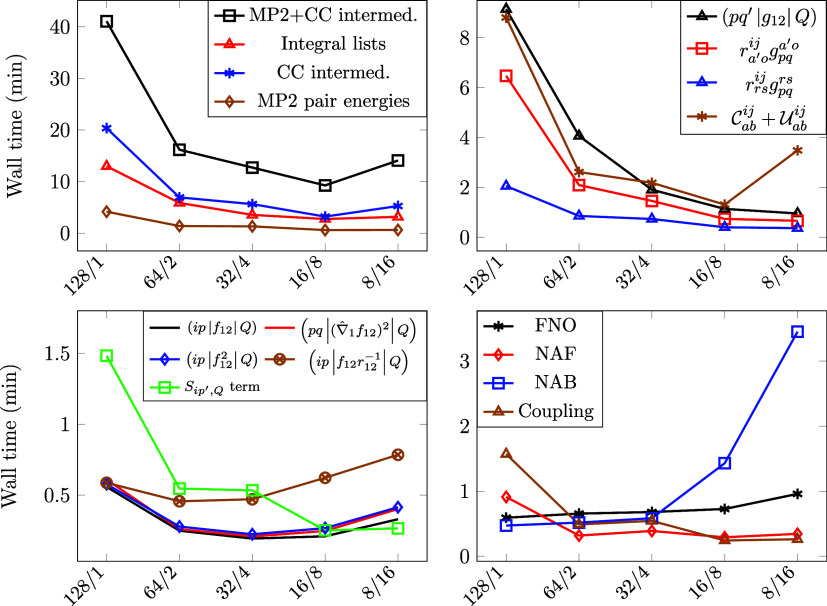
Wall times in minutes wrt. the number of MPI processes and the number of OpenMP threads for the corannulene molecule in the cc-pVDZ-F12 basis (90 active electrons, 670 active AOs) utilizing the FNO, NAF, and NAB techniques. On the horizontal axis, the number of OpenMP threads per MPI process is indicated by the first number, while the number of MPI processes per node is indicated by the second one after the forward slash character.

**Figure 9:**
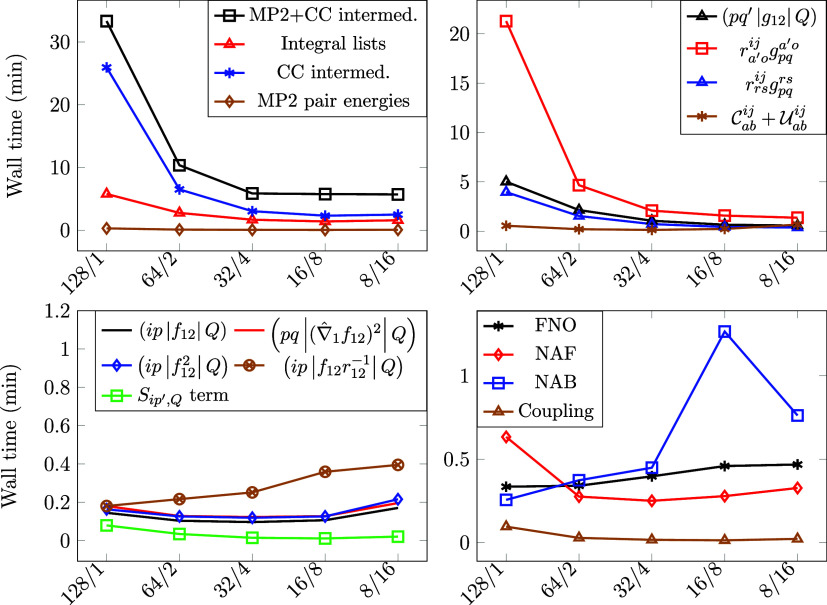
Wall times in minutes wrt. the number of MPI processes and the number of OpenMP threads for the benzene molecule in the cc-pVQZ-F12 basis (30 active electrons, 720 active AOs) utilizing the FNO, NAF, and NAB techniques. See the caption of Figure 8 for further details.

Finding the best combination in terms of the number of OpenMP threads and MPI processes for a given total number of CPU cores is a challenging task. The optimum depends, e.g., on the size of the operands in matrix–matrix multiplications, memory concurrency, and broadcast data volume. To shed light on the connection between the overall parallel efficiency and the number of OpenMP threads and MPI processes, we report measurements for a smaller and a larger molecule in Figures 8 and 9. One of the motivations is to find optimal OpenMP and MPI resource allocations for a fixed number of total available CPU cores leveraging OpenMP/MPI hybrid parallelism. In general, the execution time tends to decrease as the number of OpenMP threads increases until the MPI scaling starts to deteriorate, leading to a region of optimal OpenMP-MPI setting combinations.

Let us first discuss the case of the corannulene molecule, which is a polycyclic aromatic hydrocarbon (C_20_H_10_). For this system, with 90 active electrons and 670 active AOs in the cc-pVDZ-F12 basis, the size of (*pq′*|*g*_12_|*Q*) takes up roughly 35GB of memory, and the other integral lists require about 81GB. The computation times for various divisions of the total 128 cores to OpenMP threads (128, 64, 32, 16, 8) and MPI processes (1, 2, 4, 8, 16) are plotted in Figure 8. The total wall clock times of the F12-dependent parts (top-left, squares) range from 9.3 to 41.1 min, with the fastest time observed for 16 OpenMP threads and 8 MPI processes, while the slowest was for 128 OpenMP threads and 1 MPI process. For the integral lists, the wall clock times varied from 2.7 to 13.0 min, with the shortest time achieved again with 16 OpenMP threads and 8 MPI processes, and the longest with 128 OpenMP threads and 1 MPI process. It is also pleasing that the timings are similarly good for multiple combinations around the optima, making it simpler to find good parallel settings in practice.

To better understand the settings that work well, let us recall that the rate-determining terms scale very well up until about 8 MPI processes, but then, data broadcast and reduction deteriorate the parallel performance. In addition, there is a noticeable gap between 128 and 64 OpenMP threads with 1 and 2 MPI processes, respectively. This can be attributed to the fact that the nodes employed are furnished with 2 physical CPUs, each with 64 physical cores, and 4 nonuniform memory access (NUMA) domains per socket. Using 8 MPI processes results in the best resource utilization as in this case, each MPI process occupies one NUMA domain. Using 128 OpenMP threads leads to memory access concurrency, especially due to memory access latency on remote NUMA nodes. Launching 2 MPI processes with replicated storage improves both memory bandwidth and latency. In our case, we see a significant drop in wall times when OpenMP/MPI is changed from 128/1 to 64/2. Note that this tendency is prevalent for every term that we measured (see Figures 8 and 9) except for , for which the wall time is negligible compared to the total runtime.

Reassuringly, we find similar trends for the benzene molecule, having 3 times fewer active electrons and slightly larger number of AOs with the cc-pVQZ-F12 basis (Figure 9). The main difference between the cc-pVDZ-F12 and cc-pVQZ-F12 computations is the relative cost of the PPL-like terms  and  top-right panel of Figure 9) as the computational expenses of these terms stand out with the larger virtual space. Since these terms are cast as large tensor multiplications, they exhibit excellent scaling both with OpenMP and MPI. Consequently, we find a wide range of similarly optimal settings with up to 32 OpenMP threads and 16 MPI tasks, suggesting that cases dominated by the PPL-like terms could scale very well with hundreds of cores.

Considering all terms, while it is challenging to achieve efficient scaling on a large number of cores exclusively with OpenMP or MPI parallelization, their combination significantly extends the region of good scaling. The reason is that different operations are parallelized with OpenMP and with MPI, thus the two parallelization strategies can operate in synergy. In this way, for a large number of cores, one can utilize the cores that would not provide additional speedup, e.g., for an OpenMP-threaded BLAS3 operation, to work within a different MPI process, and vice versa.

### 3.6 Large-Scale Applications

**Figure 10:**
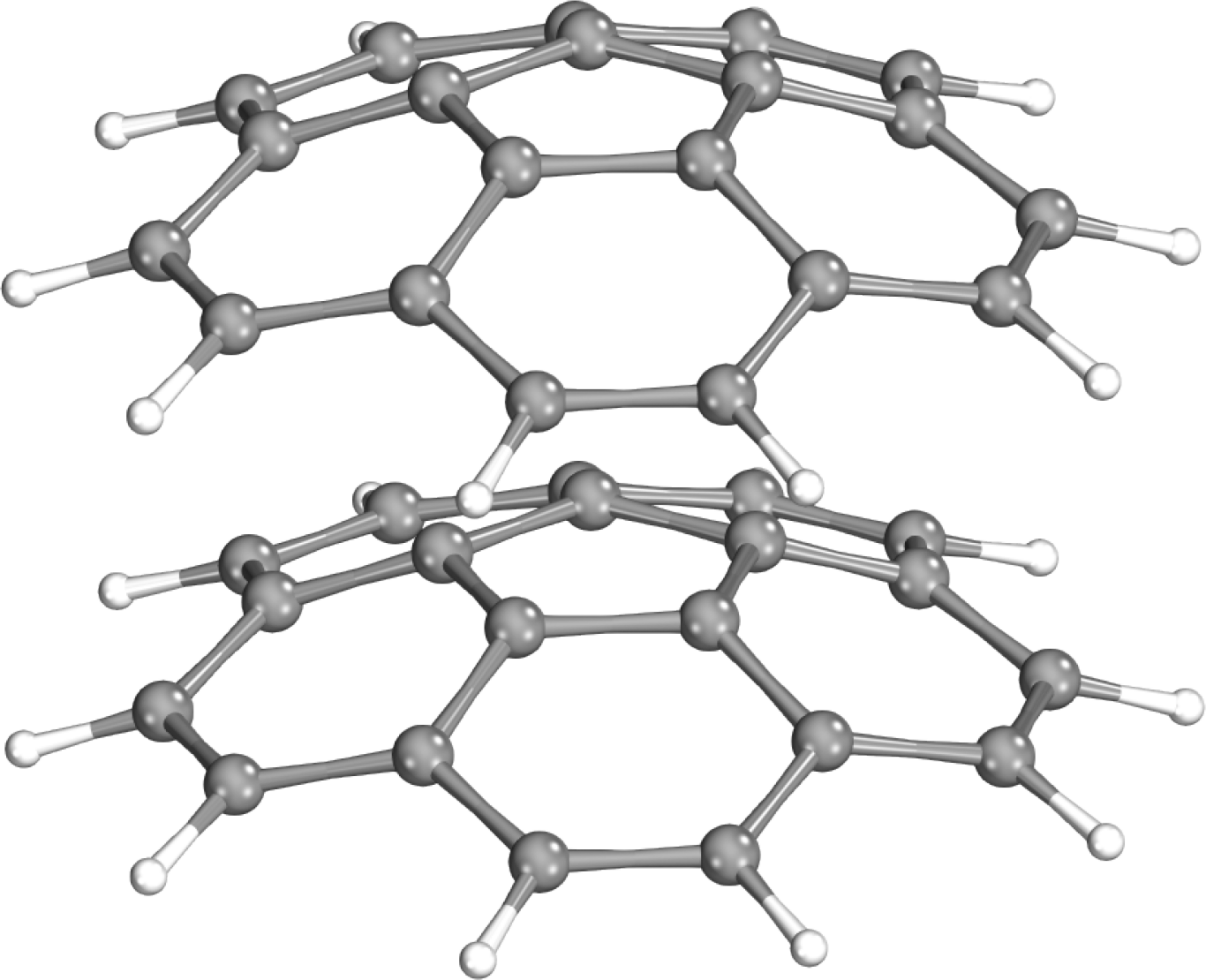
Visual illustration of the concave–convex eclipsed conformer of the corannulene dimer used for benchmark calculations in this work.

Finally, we demonstrate with large-scale applications how our parallel implementation extends the previous limits. We conducted our computations on a system with one node, featuring 16 Intel Xeon Gold CPUs (18 cores each, 288 cores total), 12 TB RAM, and a peak performance of 30 teraflops. To that end, we determine the CCSD(T)-level noncovalent interaction energy (NCIE) of the 60-atom corannulene dimer (Figure 10) close to its basis set limit. This choice is motivated by challenges uncovered by us and our collaborators to get agreement between highly regarded fixed-node diffusion Monte Carlo (FN-DMC) and CCSD(T) NCIEs for large and polarizable supramolecules with extended delocalized π-electron systems.^85^ The potential sources of the inconsistency were identified and analyzed in ref (85). both for FN-DMC (fixed-node, stochastic sampling, etc.) and for CCSD(T) (e.g., lack of higher-order correlation). Here, we can rigorously approach the basis set limit of CCSD(T) NCIEs without relying on local correlation approaches, thereby eliminating two major sources of uncertainties on the CCSD(T) side.

**Figure 11:**
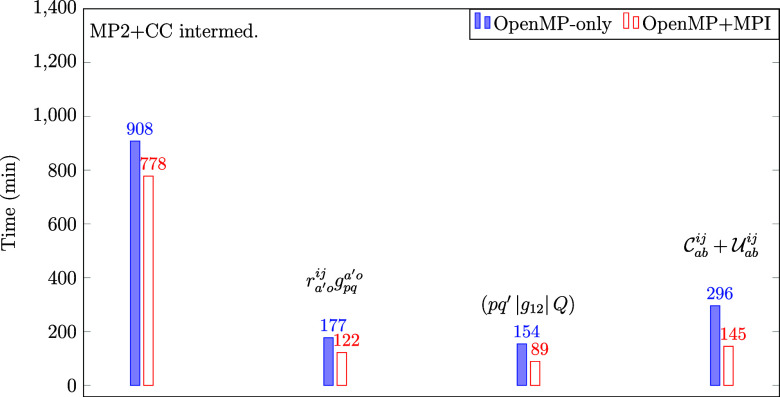
Wall times in minutes of an OpenMP-only (with 72 CPU cores) and an MPI parallel (2 processes, 36 CPU cores per process) explicitly correlated CCSD(T) calculation for the corannulene dimer using the cc-pVDZ-F12 basis set and Intel Xeon Gold 6254 processors.

Currently, our OpenMP-only implementation of the F12-dependent terms is more extensively optimized for memory consumption. For example, in the MPI algorithm, the array blocking is not fully implemented for some data-intensive parts (i.e., the entire array must be kept in memory), and a few arrays are also replicated. Therefore, it is valuable to compare both OpenMP and hybrid OpenMP/MPI parallelization for this extremely large application. Moreover, the scaling performance is expected to improve for some terms, particularly when they are processed using BLAS3 calls due to the substantially larger arrays. To demonstrate this, Figure 11 compares a 72-core OpenMP setting with a hybrid run using 2 MPI processes and 36 cores per process with the cc-pVDZ-F12 basis set. We find that the F12-dependent tasks take much less time when MPI is turned on. This is true not only for some of the terms (, (*pq′*|*g*_12_|*Q*), and  but also for the entire calculation (“MP2-F12+CC intermed.”). which is consistent with the parallel scaling analysis of Sect. 3.5.

Such large explicitly correlated CCSD(T) computations would be beyond the limits of almost all conventional implementations already with the cc-pVDZ-F12 basis set, containing 1380 AOs. The combination of hybrid OpenMP/MPI and the FNO approach also allows us for the first time to employ the cc-pVTZ-F12 basis set for such large molecules without relying on other, e.g., local correlation approximations. The cc-pVTZ-F12 basis features 2480 AOs, that is, 32% more than that of the largest FNO–CCSD(F12*)(T+) calculation performed to date,^43^ and this space is compressed to 1203 FNOs using *t*_FNO_ = 5 × 10^–5^. Having access to a relatively large amount of memory, one should opt to employ MPI parallelization. Currently, for the F12-dependent terms, the memory requirement of the MPI processes is at least 647 GB with cc-pVDZ-F12 and 2600 GB for cc-pVTZ-F12, while this could be considerably decreased to 120 and 281 GB, respectively, by using the memory-optimized OpenMP algorithm (albeit with no FNO/NAF/NAB support). Compared to that, the CCSD iterations and the (T) correction require a minimum of 111 GB with cc-pVDZ-F12 and 239 GB with cc-pVTZ-F12.

Owing to the fact that the F12 intermediates are written to the disk for the subsequent CC calculation one can stop and restart the execution once the binary evaluating them terminates. This is beneficial in a sense that we can run the explicitly correlated computation in parts, using more memory and fewer MPI processes for the F12-dependent parts and more MPI processes for the much more operation-intensive CCSD iterations and the (T) correction. The size of the different basis sets and wall times of the different calculations are presented in Table. 2. The timings for the cc-pVTZ-F12 computation are ∼5, ∼16, and ∼28.5 h for the HF iterations, the F12 intermediates, and the CCSD iterations, respectively, using 4 MPI processes and 72 CPU cores/MPI process. For the (T+) correction, we utilized 8 MPI processes and 36 CPU cores/MPI process, and the calculation took more than 5 days. More generally, depending on which bottleneck is more problematic for the given application and hardware, one can decrease the number of MPI processes or use only OpenMP to avoid memory bottlenecks for the faster F12-dependent part and use more MPI tasks and more cores altogether for the better scaling but more operation-intensive CCSD and (T) parts.

**Table 2:** Basis Set Dimensions and Wall Times [in minutes] for the Coronene Dimer Computations

basis set	*n*_0_	*n*_AO_	*n*_FNO_	*n*_NAF_	HF	MP2-F12	CCSD	(T)
cc-pVDZ-F12^a^	90	1380	888	1485	60	314	657	6863
cc-pVTZ-F12	90	2480	1203	2127	288^b^	959^b^	1713^b^	9996^c^

a 4 MPI processes and 36 OpenMP threads per process.

b 4 MPI processes and 72 OpenMP threads per process.

c 8 MPI processes and 36 OpenMP threads per process.

The NCIEs of the corannulene dimer at the HF, MP2-F12, FNO-CCSD(F12*), and FNO-CCSD(F12*)(T+) levels are presented in Table 3 both with and without counterpoise (CP)^86^ corrections. The relatively close agreement of the CP-corrected cc-pVDZ-F12 and cc-pVTZ-F12 results with each other as well as with the CP-uncorrected cc-pVDZ-F12 results is reassuring, although CP-corrected cc-pVTZ-F12 is needed to reach a few tenths of a kcal/mol uncertainty for all methods. Based on our previous benchmarks, the FNO-NAF-NAB uncertainty is expected to be similarly small.^43,68^ The new CCSD(F12*)(T+) results can be compared to the pioneering CCSD(T)/aug-cc-pVDZ computations of Janowski, Pulay, and co-workers.^87^ Although, the CP-corrected CCSD(T) results are almost identical (−14.25 vs −14.22 kcal/mol), this agreement does not hold as well for the HF, MP2, and CCSD components, indicating a potential cancellation of basis set incompleteness errors at the aug-cc-pVDZ level. Our recent FNO-CCSD(T)/def2-TZVPPD advanced the level of basis set convergence compared to aug-cc-pVDZ, especially in light of the new FNO-CCSD(F12*)(T+) results. Namely, CP-corrected FNO-CCSD(T)/def2-TZVPPD and FNO-CCSD(F12*)(T+)/cc-pVTZ-F12 agree within ca. 0.1 kcal/mol not only at the total CCSD(T), but also at the HF and CCSD levels. However, one could not assign that high level of confidence to these results when considering only the difference of the FNO-CCSD(T)/def2-TZVPPD results with and without CP corrections. The advancements of the computational infrastructure presented in this study enabling FNO-CCSD(F12*)(T+)/cc-pVTZ-F12 at this size range are very useful for obtaining basis set limit CCSD(T) results with high confidence.

**Table 3:** NCIE of the Corannulene Dimer in kcal/mol (with and without CP Corrections) Calculated with HF as well as Conventional and Explicitly Correlated MP2, CCSD, and CCSD(T) Methods

basis set	CP	HF + CABS	MP2-F12	CCSD(F12*)	CCSD(F12*)(T+)
cc-pVDZ-F12	w/o CP	13.01	–29.96	–10.25	–16.66
	with CP	14.58	–28.55	– 8.97	–15.00
cc-pVTZ-F12	w/o CP	14.20	–29.34	– 8.59	–14.53
	with CP	14.50	–28.93	– 8.23	–14.22

basis set	CP	HF	MP2	CCSD	CCSD(T)
aug-cc-pVDZ^a^	with CP	14.75	–27.25	–8.8	–14.25
def2-TZVPPD^b^	w/o CP	13.73	–36.74	–16.62	–23.68
	with CP	14.51	–28.05	–8.38	–14.30

a Taken from Table 1 of ref (87). by interpolating to the intermonomer distance of 3.69 Å and noting that CCSD(T) and QCISD(T) are almost identical for this case.

b FNO-CCSD(T) results from ref (68). with FNO and NAF thresholds identical with the ones employed here.

## 4 Conclusions

In this work, we efficiently parallelized the explicitly correlated CCSD(F12*)(T+) method and its reduced-cost, FNO-based variant using a hybrid OpenMP/MPI approach. Here, building on our previous parallel CCSD(T) code,^5^ we optimized the computationally expensive MP2-F12 part and the F12-dependent CCSD intermediates as well as the additional integral transformations required for the FNO-NAF-NAB basis set compression approximations. By mitigating these bottlenecks, the resulting conventional and reduced-cost CCSD(F12*)(T+) program can now handle almost as large systems as our efficient CCSD(T) and FNO-CCSD(T) codes.^5,68^

Undertaking such optimization is important because F12-based theories are quite complicated, and as a result, their development for modern many-core CPUs and HPC clusters lags behind advancements available for CCSD(T), for example. We have shown that the operation-intensive terms of the F12 intermediates can be formulated via efficient matrix–matrix multiplications that parallelize well up to a few dozen OpenMP threads. However, not all operations scale well with an even larger number of threads, especially for systems of moderate size and for the typically memory-bound operations of integral evaluation and transformation. To solve this, we employ hybrid OpenMP/MPI strategies. Using MPI on top of OpenMP helps scale the data-intensive operations by distributing them across different nodes and/or executing them in a shifted manner, alongside operation-bound terms.

To showcase the developments, extensive scaling measurements have been performed for typical target molecules of 12–42 atoms and double- to quadruple-ζ-F12 basis sets. These reveal excellent scaling to dozens of MPI processes for the more expensive MP2-F12 part and the (e.g., PPL-like) F12 intermediates of CCSD, while relatively poor scaling can be obtained for the less costly terms, like the transformation to the FNO-NAF-NAB basis sets. We verified for the investigated systems almost ideal scaling for the most expensive CCSD iteration and (T) correction terms. Therefore, overall, very high parallel efficiency can be achieved for the full (FNO−)CCSD(F12*)(T+) computation by combining a few dozen MPI processes with a few dozen OpenMP threads per MPI process or up to hundreds of CPU cores in total. As HPC compute nodes with 100+ cores become common and 200+ core nodes emerge, we also report scaling measurements to determine optimal parallelization settings for such machines. Encouragingly, the region of optimal performance in terms of OpenMP threads and MPI processes is quite broad.

To demonstrate the capabilities and current limits of the new FNO-CCSD(F12*)(T+) implementation, we performed large-scale calculations on the corannulene dimer up to the cc-pVTZ-F12 basis using 288 cores. At the range of 60 atoms and almost 2500 atomic orbitals, to our knowledge, this computation surpasses the previous limits of explicitly correlated CCSD(T) without relying on other, e.g., local correlation approximations. Regarding the noncovalent interaction energies, our results echo the slow basis set convergence without F12 methods, while having access to cc-pVTZ-F12 results provides confidence in the interaction energies on the scale of a few tenth of a kcal/mol. The FNO-CCSD(F12*)(T+)/cc-pVDZ-F12 result is also well within chemical accuracy of the cc-pVTZ-F12 reference.

More generally, the presented advancements are useful for multiple reasons. From the perspective of method and algorithm development, efficient and parallel explicitly correlated CCSD(T) codes are scarce, and ours in Mrcc appears to be the only one that both implements the accurate CCSD(F12*) variant and is openly accessible for academic use. Additional unique features of the FNO-CCSD(F12*)(T+) methodology, namely the FNO-NAF-NAB^43^ and the (T+)^33^ approaches, further enhance the efficiency and accuracy. Reaching approximately 60 atoms with cc-pVTZ-F12 and even larger systems with the often sufficient cc-pVDZ-F12 basis sets enables a range of advanced applications. The highly reliable (FNO−)CCSD(F12*)(T+) method can be used to benchmark lower-cost approaches, such as local CC and density functional methods. The application of (FNO−)CCSD(F12*)(T+) is recommended for medium-sized systems where local approximations are not yet effective, i.e., systems with approximately 15–25 atoms.^88^ Moreover, (FNO−)CCSD(F12*)(T+) is ideal to be part of reliable thermochemical protocols developed for medium-sized molecules of about 20–50 atoms.^89−92^

## Supporting Information Available

Geometries of the test systems and timing data plotted in Figure 7 (PDF)
