# Supplementary material for: Enabling Accurate and Large-Scale Explicitly Correlated CCSD(T) Computations via a Reduced-Cost and Parallel Implementation
Source: J Chem Theory Comput. 2025 Feb 26;21(5):2432–47. doi: 10.1021/acs.jctc.4c01777 (PMC11912216; doi:10.1021/acs.jctc.4c01777)
Supplement: Supplementary file 1 — ct4c01777_si_001.pdf [file ct4c01777_si_001.pdf]

Supporting Information: Enabling accurate and large-scale explicitly correlated CCSD(T) computations via a reduced-cost and parallel implementation

Bence Ladóczki,[*,†,‡,¶] László Gyevi-Nagy,[†,‡,¶] Péter R. Nagy,[†,‡,¶] and Mihály Kállay[*,†,‡,¶]

†*Department of Physical Chemistry and Materials Science, Faculty of Chemical Technology and Biotechnology, Budapest University of Technology and Economics, Műegyetem rkp. 3., H-1111 Budapest, Hungary*

‡*HUN-REN–BME Quantum Chemistry Research Group, Műegyetem rkp. 3., H-1111 Budapest, Hungary*

¶*MTA–BME Lendület Quantum Chemistry Research Group, Műegyetem rkp. 3., H-1111 Budapest, Hungary*

E-mail: ladoczki.bence@vbk.bme.hu; kallay.mihaly@vbk.bme.hu

Table S1: Wall times and speedups of explicitly correlated CCSD(T) calculations in minutes for various molecules in the cc-pVDZ-F12 basis set with respect to the number of MPI processes (physically separated nodes). The number of atoms is given in parentheses. The abbreviation MP2+CC int.meds. stands for the calculation of all the necessary integrals, the FNO, NAB, and NAF transformations, and the calculation of the CC intermediates. The speedup for the total calculation with respect to a single MPI process is presented in front of the forward slash character.

Molecule	MPI 1	MPI 2	MPI 4	MPI 8	MPI 16
**Nitroaldehyde product** (28)					
MP2+CC int.meds.	27.1	15.5	8.9	6.2	6.4
CCSD	35.4	19.2	10.6	6.3	4.2
(T)	123.4	61.4	30.8	15.7	9.1
Total	186.0	1.9/96.4	3.7/ 50.6	6.5/28.4	9.3/19.9
**Guanine-Cytosine** (29)					
MP2+CC int.meds.	55.5	29.6	17.0	12.0	12.9
CCSD	107.0	55.8	30.5	17.5	11.1
(T)	421.0	208.0	105.2	53.9	27.8
Total	583.6	2.0/293.7	3.8/153.0	7.0/83.6	11.2/52.1
**Tetracene** (30)					
MP2+CC int.meds.	42.7	22.8	13.1	10.4	11.1
CCSD	67.2	35.2	19.0	11.1	7.0
(T)	237.9	116.1	58.4	29.8	16.5
Total	347.9	2.0/174.3	3.8/90.7	6.7/51.6	10.0/34.8
**Corannulene** (30)					
MP2+CC int.meds.	58.8	31.1	17.7	13.4	14.8
CCSD	92.0	48.3	26.6	15.4	9.8
(T)	386.8	189.7	95.6	48.5	25.6
Total	537.7	2.0/269.2	3.8/140.0	6.9/77.4	10.7/50.3
**Penicillin** (42)					
MP2+CC int.meds.	231.5	115.1	62.0	40.5	31.2
CCSD	539.1	278.2	147.8	83.2	50.4
(T)	2893.9	1422.4	710.9	361.3	185.6
Total	3664.7	2.0/1816.0	4.0/920.9	7.6/485.3	13.7/267.4

In the following tables, we list the geometries of the molecules that we used in this work to benchmark our implementation. The coordinates are given in Ångströms.

Table S2: Benzene geometry.

Atom	x-coordinate	y-coordinate	z-coordinate
C	0.000000	-0.001000	1.070000
C	1.212436	0.000000	1.770000
C	1.212436	0.000000	3.170000
C	0.000000	0.000000	3.870000
C	-1.212436	0.000000	3.170000
C	-1.212436	0.001000	1.770000
H	0.000000	0.000000	0.000000
H	2.155537	0.000000	1.225500
H	2.155537	0.000000	3.714500
H	0.000000	0.000000	4.959000
H	-2.155537	0.000000	3.714500
H	-2.155537	0.000000	1.225500

Table S3: Cyclic dihydrooxazine N-oxide (OO) geometry.

Atom	x-coordinate	y-coordinate	z-coordinate
C	-1.186489	1.869754	-0.480728
C	-1.385452	0.424095	-0.830955
C	-0.204077	-0.455131	-0.376068
C	1.062954	0.367413	-0.561313
O	0.978827	1.529617	0.313960
N	-0.116124	2.377844	0.061805
C	-2.705367	-0.089900	-0.282311
C	-3.033843	0.098306	1.060144
C	-4.219940	-0.405250	1.574527
C	-5.097308	-1.103954	0.751810
C	-4.780917	-1.292802	-0.586545
C	-3.591527	-0.786211	-1.099063
C	-0.160023	-1.777205	-1.129999
N	2.277714	-0.305777	-0.286623
O	0.080480	3.532025	0.412998
H	1.109650	0.742500	-1.593238
H	-0.325337	-0.642012	0.695539
H	-1.441505	0.356488	-1.926545
H	-1.966456	2.596971	-0.653565
H	-3.349470	-0.933416	-2.147119
H	-5.461262	-1.832072	-1.235890
H	-6.025254	-1.494983	1.152938
H	-4.463237	-0.248731	2.619321
H	-2.356399	0.652051	1.703140
H	-1.104350	-2.314691	-1.017686
H	0.012607	-1.606903	-2.197599
H	0.648624	-2.411023	-0.763991
C	2.433027	-0.850144	1.061923
C	3.682770	-1.716913	1.104337
H	2.513165	-0.048647	1.810052
H	1.568094	-1.467713	1.313686
O	4.829985	-0.993171	0.719776
H	3.862616	-2.074231	2.119202
H	3.545014	-2.585016	0.440605
C	4.684468	-0.480347	-0.586884
C	3.473668	0.433254	-0.688649
H	5.598910	0.069842	-0.812376
H	4.578857	-1.307475	-1.305872
H	3.626508	1.321126	-0.058485
H	3.352105	0.765904	-1.723620

Table S4: Guanine-cytosine (GC) geometry.

Atom	x-coordinate	y-coordinate	z-coordinate
C	-1.598228	-2.949036	3.250000
C	-4.000540	-2.906527	3.250000
C	-4.010728	-1.569866	3.250000
C	-2.719298	-0.918718	3.250000
N	-2.830899	-3.586836	3.250000
N	-1.594926	-1.599866	3.250000
N	-2.653199	0.402428	3.250000
O	-0.598071	-3.629523	3.250000
H	-2.806641	-4.581039	3.250000
H	-4.897292	-3.497190	3.250000
H	-4.923580	-1.008975	3.250000
H	-3.479494	0.950075	3.250000
H	-1.758104	0.864659	3.250000
C	1.069242	1.108675	3.250000
C	2.285426	-1.022118	3.250000
C	3.455046	0.844410	3.250000
C	2.372412	1.689149	3.250000
C	4.058669	2.949205	3.250000
N	1.142384	-0.283906	3.250000
N	3.479383	-0.500070	3.250000
N	2.783809	3.007658	3.250000
N	4.536778	1.657659	3.250000
N	2.134562	-2.349372	3.250000
O	-0.013009	1.651379	3.250000
H	0.255022	-0.761450	3.250000
H	4.722994	3.789401	3.250000
H	5.483879	1.360580	3.250000
H	2.960976	-2.896653	3.250000
H	1.238164	-2.794426	3.250000

Table S5: Penicillin geometry.

Atom	x-coordinate	y-coordinate	z-coordinate
N	3.17265	1.15815	-0.09175
C	2.66167	0.72032	1.18601
C	4.31931	0.59242	-0.73003
C	2.02252	1.86922	-0.54680
C	1.37143	1.52404	0.79659
S	2.72625	-1.05563	0.80065
C	4.01305	-0.91195	-0.52441
C	5.58297	1.09423	-0.06535
O	1.80801	2.36292	-1.62137
N	0.15715	0.73759	0.70095
C	5.25122	-1.72918	-0.12001
C	3.41769	-1.50152	-1.81857
O	6.60623	1.14077	-0.91855
O	5.72538	1.40990	1.08931
C	-1.08932	1.35001	0.75816
C	-2.30230	0.45820	0.54941
O	-1.19855	2.53493	0.96288
O	-3.48875	1.21403	0.57063
C	-4.66939	0.59150	0.27339
C	-4.84065	-0.79240	0.11956
C	-5.79523	1.39165	0.03916
C	-6.07568	-1.34753	-0.22401
C	-7.03670	0.85454	-0.30482
C	-7.18253	-0.52580	-0.43612
H	3.24354	1.09074	2.02120
H	4.33865	0.87909	-1.77554
H	1.26605	2.42501	1.39138
H	0.17381	-0.25857	0.47675
H	6.05024	-1.64196	-0.89101
H	5.67754	-1.39089	0.85176
H	5.01118	-2.81229	-0.01401
H	2.50304	-0.95210	-2.14173
H	4.15186	-1.44541	-2.65467
H	3.14138	-2.57427	-1.69700
H	7.29069	1.46408	-0.31004
H	-2.21049	-0.02915	-0.44909
H	-2.34192	-0.28647	1.37775
H	-4.00164	-1.48999	0.26950
H	-5.69703	2.48656	0.12872
H	-6.17811	-2.44045	-0.33185
H	-7.89945	1.51981	-0.47737
H	-8.15811	-0.96111	-0.71027

Table S6: Corannulene dimer geometry.

Atom	x-coordinate	y-coordinate	z-coordinate
C	2.429314	2.165241	-0.252679
H	3.368389	2.580035	-0.612714
C	1.308567	2.979512	-0.252679
H	1.412870	4.000803	-0.612714
C	0.000000	2.480290	0.093796
C	0.000000	1.201525	0.615799
C	-1.308567	2.979512	-0.252679
H	-1.412870	4.000803	-0.612714
C	-2.429314	2.165241	-0.252679
H	-3.368389	2.580035	-0.612714
C	-2.358896	0.766452	0.093796
C	-1.142718	0.371292	0.615799
C	-3.238053	-0.323802	-0.252679
H	-4.241591	-0.107403	-0.612714
C	-2.809966	-1.641319	-0.252679
H	-3.494649	-2.406254	-0.612714
C	-1.457878	-2.006597	0.093796
C	-0.706238	-0.972054	0.615799
C	-0.692660	-3.179632	-0.252679
H	-1.208578	-4.067182	-0.612714
C	0.692660	-3.179632	-0.252679
H	1.208578	-4.067182	-0.612714
C	1.457878	-2.006597	0.093796
C	0.706238	-0.972054	0.615799
C	2.809966	-1.641319	-0.252679
H	3.494649	-2.406254	-0.612714
C	3.238053	-0.323802	-0.252679
H	4.241591	-0.107403	-0.612714
C	2.358896	0.766452	0.093796
C	1.142718	0.371292	0.615799
C	2.429314	2.165241	-3.942680
H	3.368389	2.580035	-4.302710

Table S7: Corannulene dimer geometry continued.

Atom	x-coordinate	y-coordinate	z-coordinate
C	1.308567	2.979512	-3.942680
H	1.412870	4.000803	-4.302710
C	0.000000	2.480290	-3.596200
C	0.000000	1.201525	-3.074200
C	-1.308567	2.979512	-3.942680
H	-1.412870	4.000803	-4.302710
C	-2.429314	2.165241	-3.942680
H	-3.368389	2.580035	-4.302710
C	-2.358896	0.766452	-3.596200
C	-1.142718	0.371292	-3.074200
C	-3.238053	-0.323802	-3.942680
H	-4.241591	-0.107403	-4.302710
C	-2.809966	-1.641319	-3.942680
H	-3.494649	-2.406254	-4.302710
C	-1.457878	-2.006597	-3.596200
C	-0.706238	-0.972054	-3.074200
C	-0.692660	-3.179632	-3.942680
H	-1.208578	-4.067182	-4.302710
C	0.692660	-3.179632	-3.942680
H	1.208578	-4.067182	-4.302710
C	1.457878	-2.006597	-3.596200
C	0.706238	-0.972054	-3.074200
C	2.809966	-1.641319	-3.942680
H	3.494649	-2.406254	-4.302710
C	3.238053	-0.323802	-3.942680
H	4.241591	-0.107403	-4.302710
C	2.358896	0.766452	-3.596200
C	1.142718	0.371292	-3.074200

Table S8: Corannulene geometry.

Atom	x-coordinate	y-coordinate	z-coordinate
C	2.429314	2.165241	-0.252679
H	3.368389	2.580035	-0.612714
C	1.308567	2.979512	-0.252679
H	1.412870	4.000803	-0.612714
C	0.000000	2.480290	0.093796
C	0.000000	1.201525	0.615799
C	-1.308567	2.979512	-0.252679
H	-1.412870	4.000803	-0.612714
C	-2.429314	2.165241	-0.252679
H	-3.368389	2.580035	-0.612714
C	-2.358896	0.766452	0.093796
C	-1.142718	0.371292	0.615799
C	-3.238053	-0.323802	-0.252679
H	-4.241591	-0.107403	-0.612714
C	-2.809966	-1.641319	-0.252679
H	-3.494649	-2.406254	-0.612714
C	-1.457878	-2.006597	0.093796
C	-0.706238	-0.972054	0.615799
C	-0.692660	-3.179632	-0.252679
H	-1.208578	-4.067182	-0.612714
C	0.692660	-3.179632	-0.252679
H	1.208578	-4.067182	-0.612714
C	1.457878	-2.006597	0.093796
C	0.706238	-0.972054	0.615799
C	2.809966	-1.641319	-0.252679
H	3.494649	-2.406254	-0.612714
C	3.238053	-0.323802	-0.252679
H	4.241591	-0.107403	-0.612714
C	2.358896	0.766452	0.093796
C	1.142718	0.371292	0.615799

Table S9: Tetracene geometry.

Atom	x-coordinate	y-coordinate	z-coordinate
C	0.0000	0.0000	0.7220
C	0.0000	0.0000	-0.7220
C	0.0000	1.2285	1.4004
C	0.0000	-1.2285	1.4004
C	0.0000	1.2285	-1.4004
C	0.0000	-1.2285	-1.4004
C	0.0000	2.4375	0.7213
C	0.0000	-2.4375	0.7213
C	0.0000	2.4375	-0.7213
C	0.0000	-2.4375	-0.7213
C	0.0000	3.6906	1.4026
C	0.0000	-3.6906	1.4026
C	0.0000	3.6906	-1.4026
C	0.0000	-3.6906	-1.4026
C	0.0000	4.8638	0.7120
C	0.0000	-4.8638	0.7120
C	0.0000	4.8638	-0.7120
C	0.0000	-4.8638	-0.7120
H	0.0000	1.2291	2.4824
H	0.0000	-1.2291	2.4824
H	0.0000	1.2291	-2.4824
H	0.0000	-1.2291	-2.4824
H	0.0000	3.6896	2.4834
H	0.0000	-3.6896	2.4834
H	0.0000	3.6896	-2.4834
H	0.0000	-3.6896	-2.4834
H	0.0000	5.8043	1.2417
H	0.0000	-5.8043	1.2417
H	0.0000	5.8043	-1.2417
H	0.0000	-5.8043	-1.2417

Table S10: Nitroaldehyde product

Atom	x-coordinate	y-coordinate	z-coordinate
C	-1.550470	-1.315267	-0.147114
H	-1.523342	-1.428692	-1.235474
C	-0.686247	-0.095262	0.265925
H	-0.874191	0.111413	1.324723
C	-1.108661	1.150028	-0.515355
H	-2.170690	1.369615	-0.395885
H	-0.867325	1.083580	-1.574122
C	-1.081030	-2.600158	0.536203
H	-0.065500	-2.853633	0.230554
H	-1.082779	-2.484787	1.624772
H	-1.734671	-3.437619	0.280420
C	0.798122	-0.353629	0.096242
C	1.325969	-0.701743	-1.147704
C	1.660846	-0.229846	1.180815
C	2.685735	-0.929623	-1.300138
H	0.670406	-0.800563	-2.007602
C	3.024379	-0.456152	1.030770
H	1.262843	0.057414	2.148127
C	3.539343	-0.808334	-0.208912
H	3.081354	-1.199542	-2.272543
H	3.683913	-0.352252	1.884615
H	4.602251	-0.983642	-0.327908
C	-2.989938	-1.035253	0.230310
O	-3.832455	-0.644342	-0.530892
H	-3.227309	-1.200252	1.305852
N	-0.388042	2.361006	0.001122
O	-0.419227	2.550870	1.200492
O	0.158313	3.073892	-0.809554

Table S11: Wall times in minutes with various numbers of MPI processes for penicillin. These numbers are presented as speedup values in Fig. S1.

Calculation	MPI1	MPI2	MPI4	MPI8	MPI16
MP2+CC intermed.	145.833	72.880	40.865	30.663	34.432
Integral lists	42.634	18.606	10.207	6.392	4.531
$(pq' \lvert g_{12} \rvert Q)$	25.446	10.588	5.516	2.849	1.706
$\left(pq \left\lvert (\hat{\nabla}_1 f_{12})^2 \right\rvert Q\right)$	1.806	0.677	0.433	0.347	0.302
$\left(ip \left\lvert f_{12} r_{12}^{-1} \right\rvert Q\right)$	1.822	1.143	1.030	1.247	1.211
$(ip \lvert f_{12}^2 \rvert Q)$	1.859	0.755	0.505	0.439	0.412
$S_{ip',Q}$ term	9.845	4.731	2.270	1.144	0.575
$(ip \lvert f_{12} \rvert Q)$	1.844	0.703	0.444	0.358	0.314
MP2 pair energies	22.950	11.010	5.140	2.682	1.354
FNO/NAF/NAB	14.339	8.944	5.834	9.916	16.986
Coupling	9.235	4.582	2.322	1.586	0.626
CC intermed.	65.867	34.281	19.645	11.634	11.522
$r_{a'o}^{ij} g_{pq}^{a'o}$	17.878	9.297	5.986	3.395	3.332
$r_{rs}^{ij} g_{pq}^{rs}$	4.265	2.434	1.443	0.875	1.223
$\mathcal{C}_{ab}^{ij} + \mathcal{U}_{ab}^{ij}$	26.830	13.313	6.695	3.738	4.181

Table S12: Wall times in minutes with various numbers of MPI processes for the OO molecule. These numbers are presented as speedup values in Fig. 5 in the main text.

Calculation	MPI1	MPI2	MPI4	MPI8	MPI16
MP2+CC intermed.	119.676	60.592	34.113	25.278	25.210
Integral lists	33.963	15.960	9.009	5.999	4.432
$(pq' \lvert g_{12} \rvert Q)$	21.322	8.854	4.387	2.308	1.362
$\left(pq \left\lvert (\hat{\nabla}_1 f_{12})^2 \right\rvert Q\right)$	1.241	0.757	0.579	0.512	0.491
$\left(ip \left\lvert f_{12} r_{12}^{-1} \right\rvert Q\right)$	1.285	1.057	1.020	1.202	1.110
$(ip \lvert f_{12}^2 \rvert Q)$	1.274	0.769	0.603	0.565	0.540
$S_{ip',Q}$ term	7.629	3.866	1.933	0.978	0.549
$(ip \lvert f_{12} \rvert Q)$	1.180	0.653	0.484	0.428	0.376
MP2 pair energies	17.201	8.643	4.351	2.562	1.263
FNO/NAF/NAB	12.735	7.607	4.834	5.345	10.255
Coupling	7.683	3.853	1.965	0.983	0.550
CC intermed.	55.743	28.350	15.886	11.340	9.228
$r_{a'o}^{ij} g_{pq}^{a'o}$	14.002	7.195	4.018	2.506	2.766
$r_{rs}^{ij} g_{pq}^{rs}$	11.072	5.651	2.998	1.627	1.022
$\mathcal{C}_{ab}^{ij} + \mathcal{U}_{ab}^{ij}$	21.094	10.328	5.793	4.654	3.517

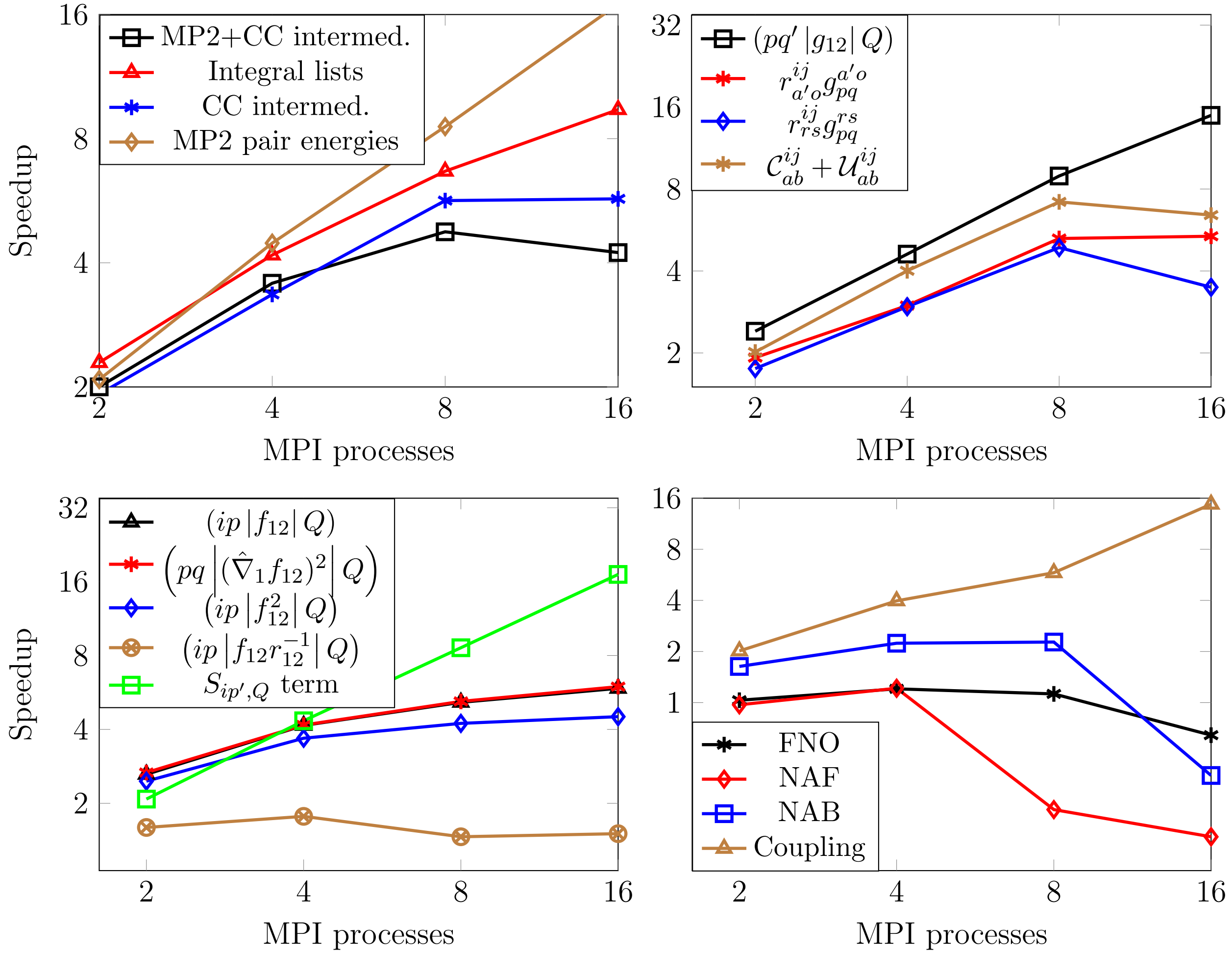

Figure S1: Speedup wrt. the number of MPI processes (16 OpenMP threads per process) for the penicillin molecule in the cc-pVDZ-F12 basis (128 active electrons, 863 active AOs) utilizing the FNO, NAF, and NAB techniques.
